# Meckel's Diverticulitis as a Cause of an Acute Abdomen in the Second Trimester of Pregnancy: Laparoscopic Management

**DOI:** 10.1155/2015/835609

**Published:** 2015-01-11

**Authors:** Ivilina Pandeva, Sumit Kumar, Atif Alvi, Hema Nosib

**Affiliations:** ^1^Department of Obstetrics and Gynecology, Hinchingbrooke Health Care NHS Trust, Hinchingbrooke Park, Huntingdon, Cambridgeshire PE29 6NT, UK; ^2^Department of General Surgery, Hinchingbrooke Health Care NHS Trust, Hinchingbrooke Park, Huntingdon, Cambridgeshire PE29 6NT, UK

## Abstract

*Introduction*. Meckel's diverticulitis is an extremely rare cause of an acute abdomen in pregnancy. Its clinical presentation tends to be rather unusual and therefore commonly delaying diagnosis. The surgical method of exploration can be either by laparoscopy or through an open incision. *Case Report*. We report a case of a 34-year-old, P1 with previous Caesarean section, who presented at 20 weeks with worsening right-sided abdominal pain, distention, and peritonism. Ultrasound scan showed an area of a possibly thickened loop of bowel inconsistent with an appendicitis. The findings at laparoscopy were purulent fluid in the pelvis, a congested appendix, and inflamed Meckel's diverticulum. An appendectomy and excision of the diverticulum was performed using stapler technique. *Discussion*. Meckel's diverticulitis in pregnancy can have nonspecific presentation and poses difficulties for preoperative diagnosis. Delay in diagnosis and management poses significant maternal and fetal risks. The use of laparoscopy if the gestational age and uterine size permit its use allows a thorough exploration of the abdominal cavity and management of rarer and unexpected pathology. Laparoscopic management of acute abdomen in the midtrimester of pregnancy has been found to be safe and effective.

## 1. Introduction

The occurrence of an acute abdomen in pregnancy is low affecting 1 : 500 [1]. Common causes are appendicitis (0.05%–0.13%) [2], followed by bowel obstruction, volvulus, intussusception (1 : 1500–1 : 3000) [3], and, even less commonly, biliary disease and pancreatitis (1 : 5000) [4].

The physiological changes in pregnancy (including nausea, vomiting, physiological leukocytosis, and increased pulse rate) together with the growing uterus pose diagnostic challenges. Delay in treatment of a surgical abdomen in pregnancy is associated with increased maternal and fetal morbidity and mortality [5]. In cases of uncertainty, a level of suspicion should be adopted, especially if radiological assessment is inconclusive. With this in mind, any progressive abdominal pain, associated with peritonism, with or without markedly raised inflammatory markers, should prompt surgical exploration.

Meckel's diverticulitis is an extremely rare cause for acute abdomen in pregnancy. Its clinical presentation tends to be rather unusual and therefore commonly delaying diagnosis.

The surgical method of exploration can be either by laparoscopy or through an open incision. The former approach is not only safe and effective during pregnancy, but also associated with good maternal and fetal outcome in the hands of experienced surgeons [6]. Other benefits include faster recovery, shorter hospital stay, and an overall better quality of life [7].

We present a case of Meckel's diverticulitis in pregnancy, which was managed laparoscopically at 20 weeks of gestation.

## 2. Case Report

A 34-year-old, Caucasian woman, gravida 2, para 1, with history of one previous Caesarean section, but otherwise low risk pregnancy, presented at 20 weeks of gestation with severe right-sided abdominal pain. She complained of a 2-day history of gradual onset pain, which was initially generalised, but localised to the right iliac fossa. Associated symptoms included nausea and diarrhoea followed by constipation.

On admission she was haemodynamically stable, apyrexial but tachycardic at 110, and tachypneoic at 24 breaths per minute. Clinical examination revealed a gravid abdomen, consistent with its gestational age, soft uterus, without Caesarean scar tenderness, and positive fetal heart rate. There were marked abdominal distension, active bowel sounds, and severe peritonism in the right flank and right lower quadrant. Blood tests showed raised inflammatory markers: leukocytosis of 13.6 × 10^9^/L, neutrophilia of 12.06 × 10^9^/L, and CRP of 207. Urinalysis was normal.

The clinical suspicion was of an acute appendicitis and a surgical opinion was sought. A subsequent ultrasound scan was performed which showed an area of a possibly thickened loop of bowel, but inconsistent with an appendicitis.

A CT scan was not performed because of the risk of fetal radiation.

The patient was kept nil by mouth and commenced on a trial of conservative treatment, including intravenous rehydration, broad-spectrum antibiotics, and analgesia. She failed to progress and was subsequently planned for a diagnostic laparoscopy +/− procedure.

A 3-port technique was used at a pressure of 12 mmHg. Findings included purulent fluid in the pelvis, a congested appendix, and inflamed Meckel's diverticulum. As a result, an appendicectomy and excision of the diverticulum was performed using stapler technique.

The fetal heart was auscultated postoperatively, and the uterus remained soft and nonirritable.

The patient made rapid recovery and was discharged home the next day following completion of 24 hours of intravenous antibiotics.

Histopathological examination of the specimen confirmed Meckel's diverticulitis.

The pregnancy continued until 39 weeks of gestation, when the patient had an elective repeat lower segment Caesarean section for previous Caesarean section and maternal request.

She was counseled thoroughly regarding the option of vaginal birth after Caesarean section; however the mother opted for a repeat Caesarean section. This decision was not influenced by her diagnosis and management for Meckel's diverticulitis earlier in the pregnancy.

## 3. Discussion

Meckel's diverticulum is a congenital abnormality. It results from the failure of the omphalomesenteric duct to involute during the 5th to 7th weeks of gestation.

It is referred to as a true diverticulum containing all layers of the intestinal wall and receives its blood supply from a terminal branch of the superior mesenteric artery. It is located at the junction of the foregut and hindgut.

The prevalence of Meckel's diverticulum is around 2% of the general population and carries 2% risk of complications. It is usually 2-3 cm in size and is twice as common in males as in females.

In the young population of less than 20 years of age, the most common complication of Meckel's diverticulum is bleeding [8]; however, after 30 years, this is replaced by bowel obstruction in 40% [9]. Other complications include intussusception (14%), inflammation (13%), bleeding (12%), and even perforation in 7%.

Pregnancy complicated by Meckel's diverticulitis is very rare. A review of the literature identified 25 cases reported worldwide and the majority of those underwent laparotomy [10–12].

The advantages of minimal access surgery in the nonpregnant population are well established and comparable in pregnant patients as well.

They include better intraoperative visualisation, less postoperative pain, faster recovery, and shorter hospital stay.

The main risk of any surgery during pregnancy is fetal loss. Most studies report on outcomes for the management of an appendicitis in pregnancy, the results of which can be extrapolated for the management of other etiologies. A review of the literature by Wilasrusmee et al. for the laparoscopic management of appendicitis versus open appendectomy reported a twofold increase in fetal loss with laparoscopic approach compared to open. This was, however, based on a single study by McGory et al. [13, 14]. There are no differences in preterm delivery rates and in fact they appeared to be slightly better in the laparoscopic group.

A benefit of the open approach is that it avoids the use of a pneumoperitoneum. But currently the effects of an increased intra-abdominal pressure and hypercapnoea to the fetus still remain unclear. The general recommendation is that the operating pressures should be kept to 12 mmHg to reduce the risks of fetal acidosis.

A review by Walsh et al. showed a 2.8% risk with the use of Veress needles for access (such as inadvertent placement of trocars into the gravid uterus, damage to the fetus) and 0% in those utilising the Hasson technique [15].

Hasson method for primary port placement in pregnancy is considered the safest. The optimum site of the primary port would depend on the size of the uterus. The umbilical and supraumbilical midline (midway between umbilicus and xiphisternum) and left upper quadrant entries have been well described.

This is then followed by the conventional 3 port placements as practiced at laparoscopy for general appendectomies. The use of single port laparoscopic surgery is still to be evaluated in pregnant population.

The mode of delivery in our case was an elective repeat Caesarean section at 39 weeks. The indication for this was the previous Caesarean section and on maternal request. This was not related to the incidence and management of Meckel's diverticulitis earlier in the pregnancy.

We feel that the mode of delivery following laparoscopic management of surgical cases of an acute abdomen in pregnancy should be based on obstetric indications.

However further research is needed to identify the effect of increased intra-abdominal pressure in the second stage of labour on recent laparotomy incision, when this is performed in the late third trimester or 6 weeks prior to delivery for the management of nonobstetric acute abdomen.

## 4. Conclusion

Laparoscopic management of acute abdomen in the midtrimester of pregnancy has been found to be safe and effective. An absolute limiting factor for performing laparoscopy in pregnancy is the enlarged uterus when it extends beyond the umbilicus, and so in the third trimester open exploration is usually the standard.

In view of the nonspecific presentation of Meckel's diverticulitis in pregnancy and the difficult preoperative diagnosis, we would recommend the use of laparoscopy if the gestational age and uterine size permit its use. This has clear benefits to the patient and would ensure a thorough exploration of the abdominal cavity and management of rarer and unexpected pathology.

## Supplementary Material

Supplementary Figure 1 showing inflamed Meckel's diverticulum.
Supplementary Figure 2 showing site of removed Meckel's diverticulum, using stapler technique.
