# Supplementary figures and images for: Meckel's Diverticulitis as a Cause of an Acute Abdomen in the Second Trimester of Pregnancy: Laparoscopic Management
Source: Case Rep Obstet Gynecol. 2015 Jan 11;2015:835609. doi: 10.1155/2015/835609 (PMC4306211; doi:10.1155/2015/835609)

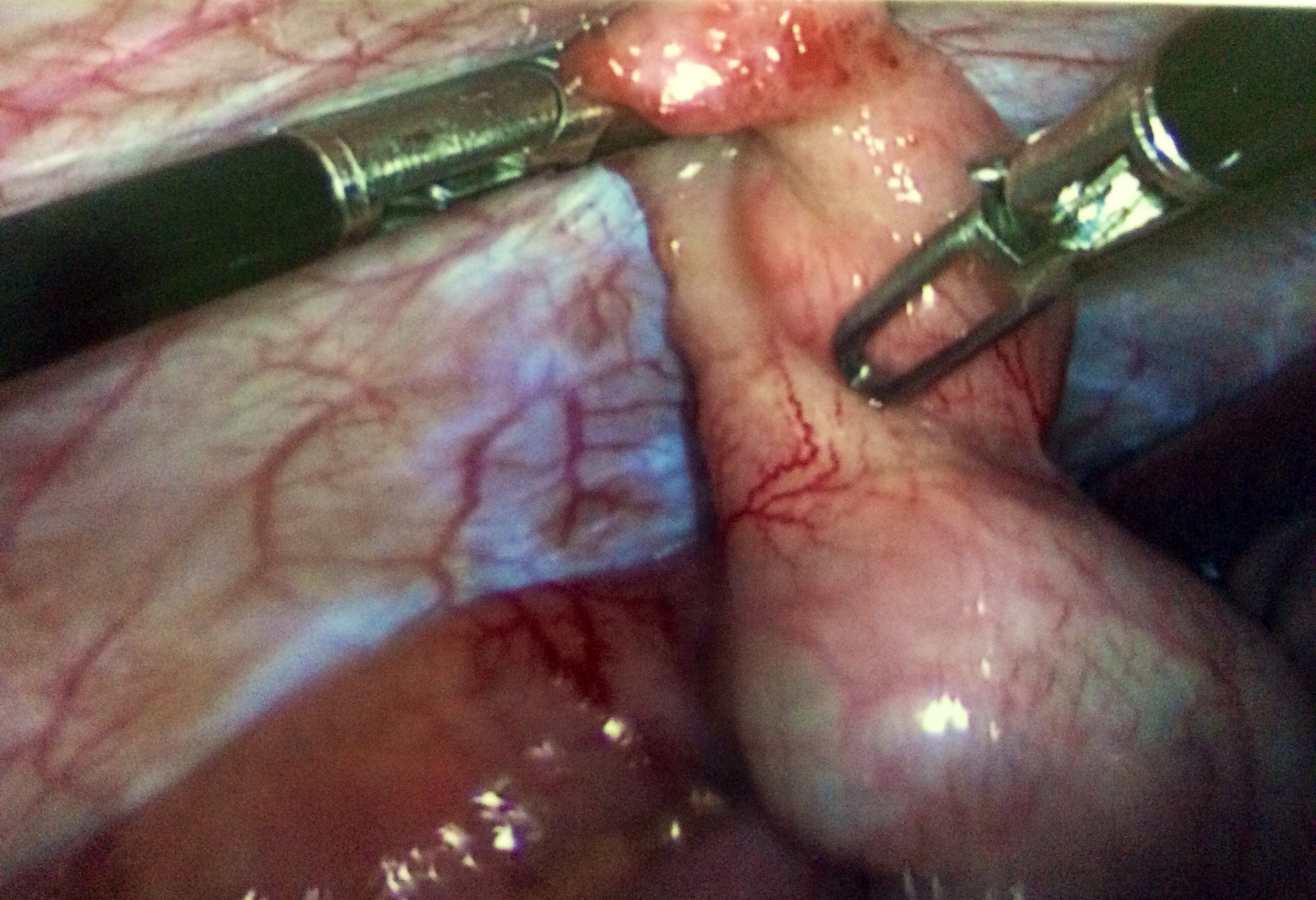

Supplement: Supplementary file 1 — Supplementary Figure 1 showing inflamed Meckel's diverticulum. Supplementary Figure 2 showing site of removed Meckel's diverticulum, using stapler technique. [file 835609.f1.jpg]
